# WWVB: A Half Century of Delivering Accurate Frequency and Time by Radio

**DOI:** 10.6028/jres.119.004

**Published:** 2014-03-12

**Authors:** Michael A Lombardi, Glenn K Nelson

**Affiliations:** National Institute of Standards and Technology, Boulder, CO 80305

**Keywords:** broadcasting, frequency, radio, standards, time

## Abstract

In commemoration of its 50th anniversary of broadcasting from Fort Collins, Colorado, this paper provides a history of the National Institute of Standards and Technology (NIST) radio station WWVB. The narrative describes the evolution of the station, from its origins as a source of standard frequency, to its current role as the source of time-of-day synchronization for many millions of radio controlled clocks.

## 1. Introduction

NIST radio station WWVB, which today serves as the synchronization source for tens of millions of radio controlled clocks, began operation from its present location near Fort Collins, Colorado at 0 hours, 0 minutes Universal Time on July 5, 1963. Thus, the year 2013 marked the station’s 50th anniversary, a half century of delivering frequency and time signals referenced to the national standard to the United States public. One of the best known and most widely used measurement services provided by the U. S. government, WWVB has spanned and survived numerous technological eras. Based on technology that was already mature and well established when the station began broadcasting in 1963, WWVB later benefitted from the miniaturization of electronics and the advent of the microprocessor, which made low cost radio controlled clocks possible that would work indoors. This paper traces the history of WWVB, beginning with the earliest radio experiments conducted by NIST’s predecessor, the National Bureau of Standards (NBS). It describes the station’s origins as a source of standard frequency, with only a small number of time users. It goes on to describe the station’s resurgence after a significant power increase in the 1990s, when it became a time-of-day synchronization source for mass produced radio controlled clocks. It then discusses recent advances that have made the station’s time signals easier to receive and more useful than ever before.

## 2. Early Radio Experiments and Frequency Measurements at NBS

In some ways, the origins of the National Bureau of Standards (NBS), now known as the National Institute of Standards and Technology (NIST), coincide with the origins of radio. NBS was founded in March 1901, just nine months before Marconi transmitted the letter “S” across the Atlantic by wireless telegraphy [1]. NBS scientists began conducting radio experiments shortly after the agency was founded, making perhaps their first contribution to the radio science literature in 1905, when Louis Austin published a paper about electrolytic detectors [2]. Austin went on to publish nearly 70 papers about radio propagation at the Bureau before his death in 1932 [1].

NBS was, of course, a standards organization. As a result much of its early radio research was focused on developing measurement standards. John H. Dellinger, who joined the Bureau in 1907 and remained there until 1948, was a pioneer in the standardization and measurement of radio frequency. In 1911, Dellinger calibrated a wavemeter sent to NBS by a wireless company [3], a task that he later called “the first radio job of the National Bureau of Standards” [1]. Dellinger obtained the frequency reference for his wavemeter calibrations from a simple calculation of the resonance of an inductance-capacitance (LC) circuit [4]. During the next decade, frequency measurement technology based on wavemeters continued to improve under Dellinger’s guidance, due to better mathematical derivations for inductance and capacitance and better measurements of capacitors and their materials. However, the uncertainty of measurements made with these wavemeter instruments was never much better than 0.1 %.

The lack of a way to accurately measure frequency was an obvious problem for the radio industry. Until it was solved, this was one of the great measurement problems of the 20th century. The new radio receiving sets being manufactured in the 1920s had to be able to accurately “tune” to the assigned frequency of the station that the listener wanted to hear, so the markings on the radio dial had to correspond to the frequencies actually being transmitted. More importantly, transmitters had to stay on their assigned frequency, because any station that strayed from its frequency assignment would interfere with other stations. By 1923, 570 U. S. broadcast stations had crowded into a 1000 kHz wide band and there was no satisfactory method for measuring the frequency of radio transmitters [1]. Dellinger explained the problem in an aptly titled paper called “Reducing the Guesswork in Tuning”:

“*The waves used by the broadcasting stations are spaced 10 kilocyles apart (3 meters at a wavelength of 300 meters). Thus one station is on 990 kilocycles, another on 1000, and another on 1,010 kilocycles. A variation of the frequency of 1 per cent, for example, would be a variation of 10 kilocycles and could cause one station to be using exactly the wave that had been assigned to another. The whole success of American broadcasting is thus tied up with the placing of broadcasting stations on the correct frequencies to an accuracy approaching 99.9 per cent. Since receiving sets are now available by which an individual can hear the stations from all over the United States on the same night, the importance of this accuracy is apparent.”* [5]

The “99.9 per cent” accuracy requirement, which wavemeters could barely meet, would soon become much more stringent. In 1927, the U. S. Congress formed the new Federal Communications Commission (FCC), which soon required all radio broadcasters to stay within 500 Hz of their assigned frequency. The requirement was tightened to 50 Hz in 1932 (5 × 10^−5^ at 1000 kHz) [1]. Even so, the problem of measuring radio frequency was largely solved by the invention of the quartz oscillator, which became commercially available in 1924 [6], and through the efforts of NBS, which had begun broadcasting standard frequency signals from a new radio station named WWV in 1923.

## 3. Radio Station WWV and its Impact on the Early Days of Radio

**Fig. 1:**
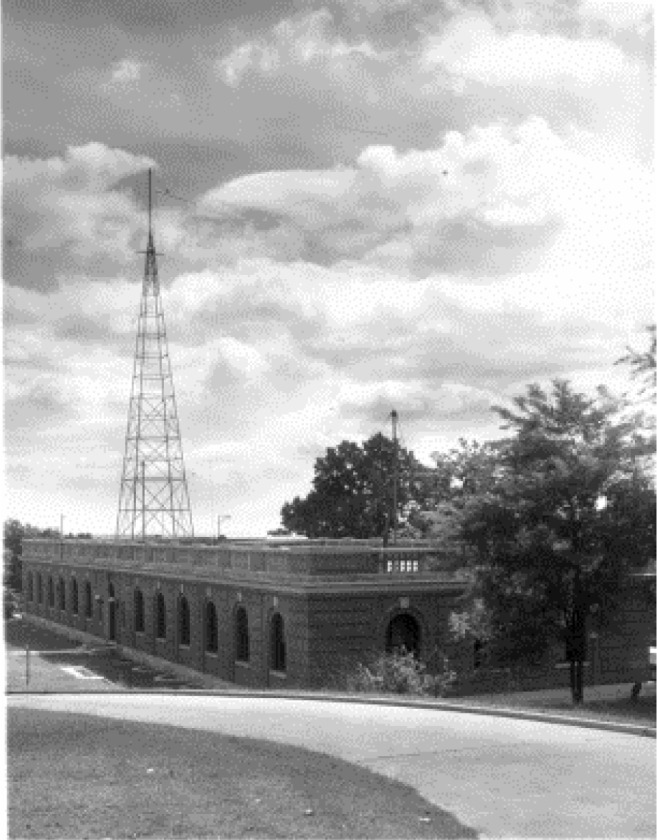
Original WWV antenna, Washington, DC.

Dellinger became the director of the radio section at NBS in 1919 [3]. In October of that year, the call sign WWV was assigned to NBS. The call letters have become synonymous with the broadcasting of time signals, but it is unknown why they were originally chosen or assigned [7]. Dellinger and his staff assembled WWV as an experimental radio station operating from the first floor of a building on Connecticut Avenue in Washington, DC in 1920 [3]. Throughout 1920 and 1921, the station was used for a variety of purposes, including the broadcasting of music concerts, and for the transmission of market news intended for farm bureaus and agricultural associations. However, it was eventually decided that the station’s primary purpose would be to transmit standard frequency signals, as a reference for other radio broadcasters. The stations first tests as a standard frequency station were conducted on January 29, 1923 [7]. Figure 1 shows part of the original WWV transmitting antenna, located on the Bureau of Standards grounds in Washington, DC.

By March 1923, WWV was broadcasting standard frequencies, ranging from 75 to 2000 kHz on a weekly or monthly schedule, using a 1 kW transmitter. In April 1923, NBS published a broadcast schedule, *Letter Circular 92*, indicating that WWV was “conducting a series of transmissions of signals of known frequency (wave length) to be used as a basis for adjusting and calibrating radio apparatus” [8]. The radio world reacted favorably. In 1924, Hoy J. Walls, an NBS physicist who helped design the station, published an article in the amateur radio magazine *QST* that described the antennas and transmission equipment. According to a *QST* editor:

*“Probably no radio station has ever rendered the American radio world so great a service as that of WWV in transmitting the standard wave signals. Before these signals began both broadcast and amateur waves were uncertain and often wavemeters disagreed violently. Since the signals began those in the East have been able to make precision calibration on their own wavemeters and to pass the information along to the West.”* [9]

The phrase “those in the East” referred to NBS personnel who adjusted the WWV transmitter to the correct frequency by using a wavemeter that had been calibrated against the national standard wavemeter. This situation changed in 1927, when the first in a series of quartz oscillators became the national standard for frequency and was installed at the station. During the transmissions, the frequency of the transmitter was adjusted manually until no beat note was audible between the transmitter and the quartz standard. Within the span of a few years, quartz standards and better measurement techniques had improved the frequency accuracy by several orders of magnitude, from parts per thousands (10^−3^) to better than one part per five million (2 × 10^−7^) by the early 1930s [10].

WWV moved to the high frequency (HF) bands, better known as shortwave, when it began a 5 MHz broadcast in 1931, and soon expanded to transmitting on multiple frequencies. It currently broadcasts continuously on five shortwave frequencies: 2.5, 5, 10, 15 and 20 MHz. WWV became a standard of time interval as well as frequency in 1937, when the station began transmitting pulses at one second intervals [11]. Ironically, the pulses were not held in phase with any time reference, because the United States Navy still served as the nation’s official radio time broadcaster. This changed in June 1944, when the Superintendent of the United States Naval Observatory (USNO) authorized the synchronization of the WWV time signals to those of the USNO, largely because the Navy had ordered the USNO to cut back time transmissions during World War II. As a result, WWV began sending time messages in telegraphic code every five minutes in October 1945 [12]. The iconic voice announcements of time, now known to nearly all who listen to shortwave radio, began on January 1, 1950 [13]. A sister station, WWVH, began operation in Hawaii in 1948 [14], adding its own voice announcements of time in 1964.

WWV and WWVH are limited as a source of accurate frequency due to the instabilities of HF radio signals as they propagate across the Earth. When WWV began transmitting in the 1920s, the uncertainties of the wavemeters that were used to calibrate the station were much larger than the uncertainties introduced by the variations in HF radio propagation. This remained true in the early days of quartz oscillators. However, as quartz oscillator technology continued to improve, it became evident that HF radio was limiting the accuracy of the frequency and time signals being delivered. This became more and more obvious as the years went on. By the 1960s, atomic oscillators were controlling the transmitted WWV and WWVH frequency to within 1 part per trillion (1 × 10^−12^), but the frequency recovered by users of the signals was much less accurate, typically by at least several orders of magnitude.

The main problem with using a HF radio signal as a frequency reference was due to changes in the height of the ionospheric layer, which caused fluctuations in the transit period of the signal. These fluctuations produced a Doppler effect that made the received frequency higher or lower than the transmitted frequency. To reduce the Doppler effect, John Shaull of NBS wrote in 1950 that “high-accuracy measurements of frequency in terms of WWV should be made when ionospheric layer heights are likely to be most stable, i. e., with noon or midnight prevailing at about halfway between transmitter and receiver locations” [15]. However, even if these instructions were followed, it was difficult to recover frequency from WWV with an uncertainty of better than parts in 10^9^, unless the user was located very close to the station and could receive a ground wave signal [15], or unless measurements were recorded and averaged over multiple days [16]. These same limitations apply to WWV measurements today.

**Fig. 2:**
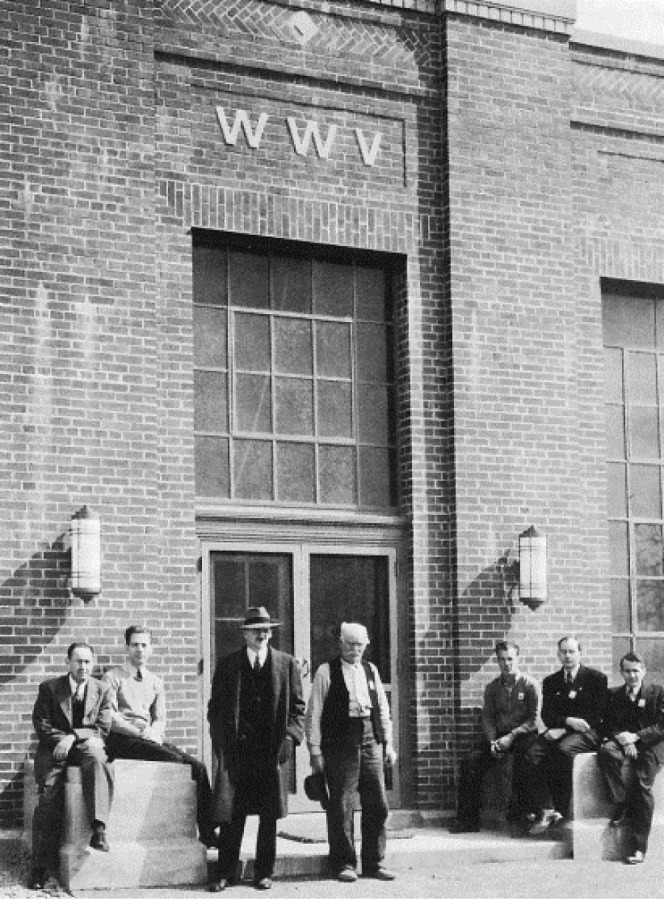
WWV transmitter building, Beltsville, Maryland, 1943.

Some nine decades after its first broadcast, WWV continues to serve both the radio and metrology communities, although it has changed locations several times. WWV moved from Washington, DC to College Park, Maryland in 1931, and then from College Park to Beltsville, Maryland in December 1932. A fire destroyed the station on November 6, 1940, but WWV was back on the air in an adjacent building just five days later. Congress provided the funds to construct a new station just 5 km to the south of the former site, which went on the air in January 1943 (Fig. 2). The station remained at the Beltsville location (later referred to as Greenbelt, Maryland) until 1966, when it was moved to Colorado [17] to share the site occupied three years previously by a new station called WWVB, as we shall see in the following sections.

## 4. CRPL and the Quest for Higher Accuracy Frequency and Time Signals

In the summer of 1942, in the midst of World War II, the Interservice Radio Propagation Laboratory (IRPL) was established at NBS to serve the U. S. military. In 1946, when the war had ended, IRPL became the Central Radio Propagation Laboratory (CRPL), and no longer had a purely military function. In 1954, the CRPL was relocated from Washington, DC to the new NBS laboratories that had just been constructed in Boulder, Colorado [1].

Upon its arrival in Boulder, one of the CRPL’s goals was to distribute the national standard of frequency at lower uncertainties than were possible through the shortwave broadcasts of WWV and WWVH. This would allow frequency standards, which were rapidly becoming more stable and accurate, to be measured faster, without the need to average data for days or weeks. Based on propagation research conducted at NBS and elsewhere, it was already known that this could be done by broadcasting a groundwave signal that traveled within the duct formed by the ionosphere and surface of the Earth, instead of relying on reflection from the ionosphere for propagation as the shortwave stations had done. Eliminating the error producing reflections from the ionosphere would greatly increase accuracy, but would require a lower transmission frequency [18].

Other low frequency (LF, 30 to 300 kHz) and very low frequency (VLF, 3 to 30 kHz) radio stations designed for measurement and calibration purposes had already been built by the time CRPL moved to Boulder. The National Physical Laboratory (NPL) of the United Kingdom had installed a high-quality quartz frequency standard at the Rugby site operated by the British Post Office. In 1950, radio station MSF began broadcasting standard frequency signals from Rugby at a carrier frequency of 60 kHz. In 1954, the same frequency standard became the reference for the 16 kHz signals broadcast from the VLF station GBR, also located at the Rugby site [19, 20]. J. A. Pierce at Harvard University in Cambridge, Massachusetts received the GBR signal and made frequency measurements across a transoceanic path of 5180 km. The measurements were accurate to about 1 × 10^−9^ over an interval of just a few minutes. Pierce noted that these results were at least two orders of magnitude better than similar frequency measurements made over a short interval with WWV [19]. It was clear that LF radio had many advantages over shortwave for the purposes of distributing frequency. In 1956, NBS began its own LF radio experiments from an experimental radio station located at its new Boulder campus.

## 5. Experimental Radio Station KK2XEI

**Fig. 3:**
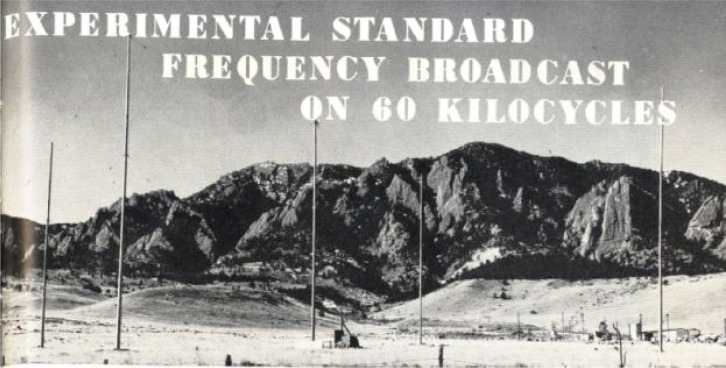
KK2XEI antenna in Boulder, Colorado.

On July 1, 1956, an unusual antenna located on the Boulder Laboratory campus, about a mile east of the mountain ridge known as the Flatirons, began radiating a 60 kHz carrier frequency that was referenced to the United States Frequency Standard. The antenna consisted of four 38 m tall poles arranged in a square, with an identical pole in the center (Fig. 3). Wires were run up the center pole, and connected at the top to other wires which terminated at each corner pole. A hut near the center pole contained tuning equipment. The antenna was connected to a 2 kW transmitter 1000 m away. The control frequency was fed to the transmitter via a 500 m transmission line. The new experimental radio station was identified by the call sign KK2XEI [21]. W. D. George, then the acting chief of the Radio Standards Laboratory, initiated the plan for the experimental broadcasts. In May 1957, George presented a paper about KK2XEI at the *11^th^ Annual Frequency Control Symposium* in Fort Monmouth, New Jersey [22]. George, who had begun his career at NBS in 1930, noted in his presentation some of the advantages that LF and VLF radio had over the existing HF signals being broadcast by NBS:

“In the 1930’s the NBS considered VLF before adoption of the plan to broadcast several high frequencies simultaneously. The principal reason for adopting the high-frequency system was the existence, at that time, of suitable receiving equipment in the hands of the public for the high frequencies and the lack of it for the low frequencies. It was recognized that the principal advantage of the low frequency would be freedom from transmission vagaries and that two outstanding disadvantages would be high antenna cost and possible interference from natural noise.

VLF may now be used; the only disadvantage appears to be the initial expense of an efficient antenna. Natural noise is overcome by reduced bandwidth receivers, coherent detectors, and integration type measuring techniques. Receivers can be built for either VLF or HF at approximately the same cost.

*The principal reasons for studying a VLF standard frequency service are to give the users an opportunity to obtain a quicker and more accurate frequency and phase-reference than is now possible and to make measurements versus a reference which may be reliably received throughout the world.”* [22]

As an experimental station, KK2XEI was typically operated from 1530 to 2000 UTC Mondays through Fridays. Since the top-loaded monopole antenna was very small electrically at 60 kHz, it was inefficient and the radiated power was less than 2 W. Despite the low power, Pierce was able to receive KK2XEI at Harvard shortly after the station went on the air in the summer of 1956. By now, Pierce was adept at using the frequency recovered from incoming LF and VLF radio signals to measure local oscillators. Shortly afterwards, he began using the radio references to control the frequency of a local oscillator, perhaps the first examples of the disciplined oscillator instruments that meet the needs of so many frequency control applications today [23]. In January 1957, Pierce measured the received frequency of KK2XEI at Harvard by comparing it to the received frequency of GBR. The differences, recorded only on weekdays only when the station was operational, ranged from −0.3 to −3.7 parts in 10^9^. The received signal was simultaneously compared to the NBS standard in Boulder, with the frequency difference never exceeding 0.8 parts in 10^9^ [22].

In February 1958, Pierce’s laboratory at Harvard obtained a cesium standard from a company called Atomichron^1^. Cesium standards represented the brand new “atomic clock” technology, which later became, as it remains today, the basis for the International System (SI) second, and Atomichron was the first company to sell cesium standards commercially [24]. NBS had already obtained two Atomichron standards [25], and one of them (Atomichron S106) was ordinarily used to calibrate a quartz oscillator that served as the frequency reference for KK2XEI. For a period of eight weeks (March-April 1958), an arrangement was made where the KK2XEI frequency was directly controlled by S106 for two days each week. This allowed the Atomichron at Harvard (S112) to be directly compared to S106 at NBS, by using KK2XEI as a transfer standard. During the last 10 days of the comparison (over a five-week period), the largest frequency difference recorded was 3 × 10^−11^. Pierce wrote, “Although it is perhaps a coincidence that the two Atomichrons were in such excellent agreement, the remarkably small scatter over a period of five weeks is an excellent testimonial to the stability of low-frequency transmission in the daytime” [26]. The experiment indicated that the previous LF experiments had probably been limited by the instabilities of quartz oscillators, and that the performance advantage that LF radio had over shortwave was even larger than previously believed. LF radio had established itself as the lowest uncertainty method for frequency transfer, a distinction it would hold until the second half of the 1970s, when it was surpassed by satellite systems.

In March 1960, the call sign of KK2XEI was changed to WWVB [27]. Although the selection of the call sign WWV for the original NBS radio station is believed to have been arbitrary [7], the “B” in WWVB likely was intended to designate the location of the original transmitter site in Boulder, just as the “H” in WWVH likely was intended to designate Hawaii. WWVB would remain in Boulder as an experimental station for several more years, while NBS staff worked on the design of a new VLF station at 20 kHz, and on the acquisition of a permanent site for all of its radio stations.

## 6. WWVL: The Quest for a Global Transmitter

The success of Pierce’s research using the 16 kHz GBR standard frequency broadcast from Rugby, England led him to believe that a single transmitter could provide accurate frequency information to the entire world. The signal would have to be in the VLF band where attenuation was much less of a problem than at the 60 kHz frequency of WWVB. Even so, the station would still require a lot of power. Pierce estimated, for example, that about 500 kW of radiated power from an antenna with an effective height of 200 m would be required to exceed natural atmospheric noise levels at all locations (a field strength of about 10 µV per meter) and provide a standard frequency service to the entire world at 20 kHz. At 10 kHz, the power required would be considerably less, only about 20 kW [23].

NBS staff members embraced Pierce’s idea of a single VLF transmitter that provided worldwide coverage. In his 1957 presentation, George had indicated the likely frequency of such a transmitter would be 10 kHz [22]. However, a comprehensive 1958 study by A. D. Watt and R. W. Plush of the NBS Boulder laboratories examined a variety of factors other than radiated power requirements, including an analysis of the carrier-to-noise ratios required for accurate frequency measurements, and recommended the use of 20 kHz [28]. As a result, plans were made for another experimental station near Boulder, this one operating at 20 kHz.

**Fig. 4:**
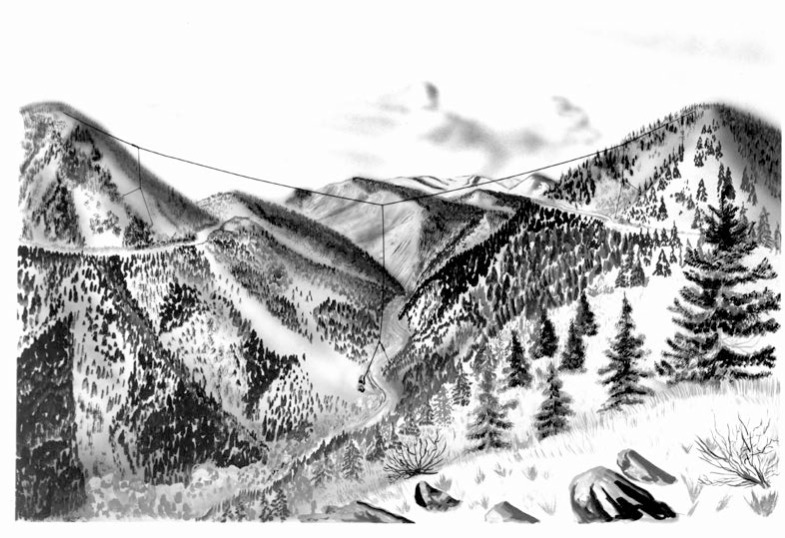
Artist’s conception of the original WWVL antenna in Fourmile Canyon, west of Boulder.

NBS had previously installed a valley-span antenna in Fourmile Canyon approximately 18 km from Boulder (near the community of Sunset, Colorado) for VLF receiving experiments. After modifications for broadcasting, this large antenna consisted of a 1036 m copper-coated steel cable suspended from two mountain peaks on opposite sides of the canyon. The cable weighed more than one ton. Another cable extended down from the center of the cross-span about 275 m to the canyon floor [29]. Figure 4 shows an artist’s view of the WWVL antenna, looking toward the west. Despite its large physical size, the antenna was quite small electrically, because the wavelength at 20 kHz is 15 km. An 8 kW transmitter was used, but the radiated output power was only about 14 watts, an efficiency of only 0.18 % [30].

The experimental station began transmitting for six hours per day on April 5, 1960 with the call letters WWVL. The “L” in the call sign did not refer to the station’s location, but was probably intended to refer, along with the “V” that preceded it, to “very low” frequency. In June 1960 the broadcasts became continuous, except for periods during the “dark” phase of the moon when another project used the antenna during the nighttime hours. Under ideal conditions, the signal could travel a great distance. Intermittent reception was reported as far away as New Zealand [30].

## 7. Selection of a Permanent Site for WWVB, WWVL, and WWV

By 1960, it was estimated that more than 50 organizations within the United States were using the experimental WWVB broadcasts, and the signals were considered reliable and accurate enough for some organizations to cancel their plans to acquire expensive atomic frequency standards [29]. The success of the experimental broadcasts at both 60 kHz and 20 kHz had made it obvious that a broadcast facility was needed where higher power transmission equipment could be permanently installed, and the search had begun for a suitable location. It was clear that the Boulder site where WWVB was currently located was too small for a high power station. It was also too close to the Rocky Mountains, which could affect the signal path to the west.

A memorandum of site considerations, dated July 6, 1959, and updated several times during the following months, listed the requirements for the new home of WWVB and WWVL. The first approach was to study maps of U. S. government and state owned land. About 20 possible sites were considered, but none were found suitable due to various problems, including unlevel ground, the risk of cyclones, poor ground conductivity, or a location that was considered to be too far away from the Boulder laboratories. Ten more sites located in a north-east-south quadrant, with the Boulder laboratories in the center, were then considered, but the soil conductivity of these sites was considered to be poor. Finally, seven sites north of Fort Collins, Colorado were considered. This region has numerous lakes and reservoirs, which was favorable for good soil conductivity. The investigated sites were in the optimum region between the mountains to the west and the cyclone region of the eastern Colorado plains. The conditions were very favorable, and it was likely determined well before the end of 1959 that the area north of Fort Collins would become the home of the new NBS radio stations.

By systematic evaluation, one of the seven sites north of Fort Collins was selected in December 1960. The selected site was a tract of farmland bordered by two reservoirs, North Poudre Reservoir 6 and Greenwalt Lake. The ground conductivity of the soil at the site was unusually high, which is desirable for the grounded portion of large antenna systems [31]. The site was located some 10 km north of Fort Collins, and about 80 km north of Boulder, which was considered far enough away to prevent the strength of the 20 kHz and 60 kHz signals from interfering with Boulder radio experiments. However, it was considered to be near enough to Boulder for the driving distance between the station and the laboratory to be reasonable, and for the station frequency to be controlled by or phase locked to the Boulder standard [32].

The land area was approximately 380 acres. It was purchased in two parcels, a 365.7 acre parcel obtained through eminent domain from Milo and Lucille Sabin at a cost of $72,500 and a 14.25 acre site obtained in the same fashion from Willard Stovall at a cost of $3,500. Although construction on the site began in the summer of 1962, the final settlement of the land purchases was not completed until March 1963.

The land cost represented less than 20 % of the total cost of building the initial configuration of the stations. Congress appropriated $325,000 for the stations in 1961, and additional funding of about $100,000 was provided by the National Aeronautics and Space Administration (NASA) [33].

## 8. The Global 20 kHz Transmitter: A Plan That Never Materialized

**Fig. 5:**
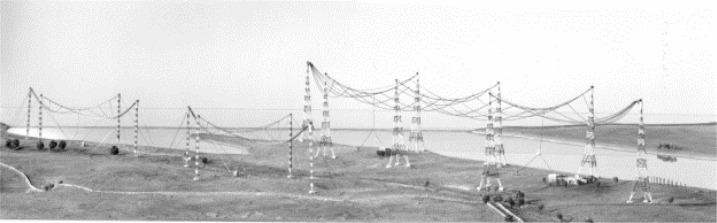
Model of the Fort Collins, Colorado radio station, with the proposed high-power VLF time and frequency antenna on right.

During the acquisition and planning stages for the new Fort Collins site, the grand vision of providing a worldwide time and frequency service from just one station persisted. According to this plan, the first stations activated at the Fort Collins site would be known as the “pilot stations”, proof of concept experiments that would pave the way for the next step, a massive eight-tower “triatic” antenna array with a 500 kW transmitter. The planned station would broadcast a 20 kHz frequency standard that could be received worldwide. The antenna was a system of cables suspended from eight 183 m high self-supporting towers, based on the design of an existing U.S. Navy antenna array in Annapolis, Maryland. The projected efficiency of the antenna was 33 %. A photograph of a model of the proposed station is shown in Fig. 5 [34].

**Fig. 6:**
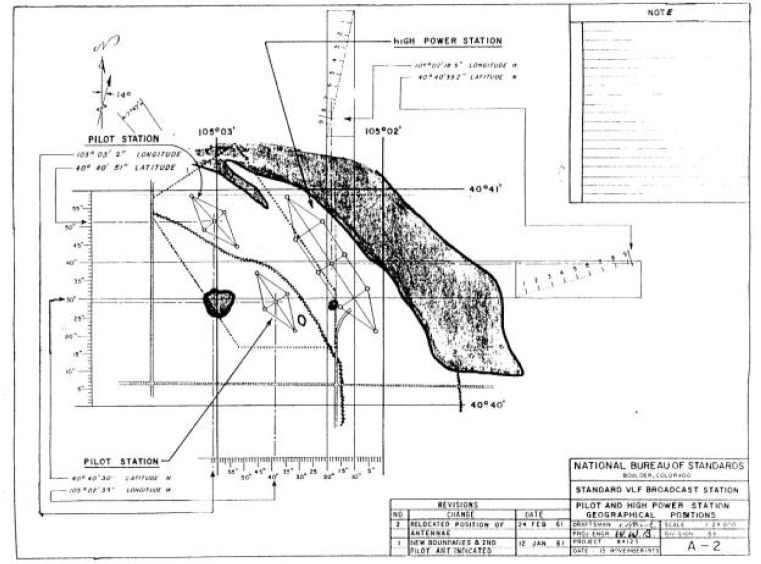
Fort Collins antennas, including the proposed high power VLF station antenna.

The plans for the new site also included new facilities for WWV; the HF antennas were shown on the model as short sticks (lower right of Fig. 5). An engineering drawing showing the locations of the antennas is shown in Fig. 6. However, fiscal, political, and technical circumstances intervened, and the large VLF antenna array was never built.

## 9. The Original Station Design and the First Broadcasts from Fort Collins

**Fig. 7:**
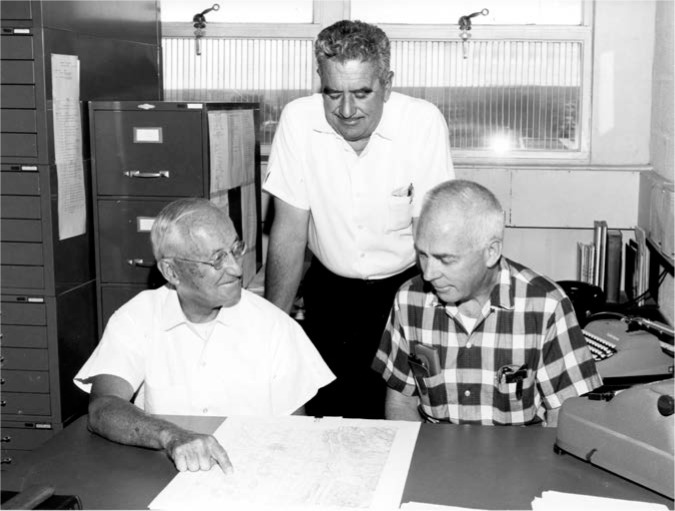
NBS staff members in Boulder discussing plans for the new stations in Fort Collins (from left to right, W. W. Brown, Richard Carle, and Al Morgan).

Plans did move forward on the pilot stations, which would become the main broadcast facility. The stations were designed by W. W. Brown, an antenna engineer who had been heavily involved in the selection of the Fort Collins site. Brown had a distinguished 44-year career designing LF and VLF transmission systems at the General Electric Company before retiring and coming to NBS in 1958, where he would retire for a second time in 1965. It is interesting to note that a 1926 paper by Brown shows an antenna that bears a remarkable resemblance to the antennas that would be built in Fort Collins [35]. Figure 7 shows Brown (left), Richard Carle (standing, middle), and Al Morgan (right), discussing the station plans in Boulder shortly before the opening of the Fort Collins site. Carle was the original engineer-in-charge (EIC) of the Fort Collins site, and Morgan was the chief of the radio broadcast section.

**Fig. 8:**
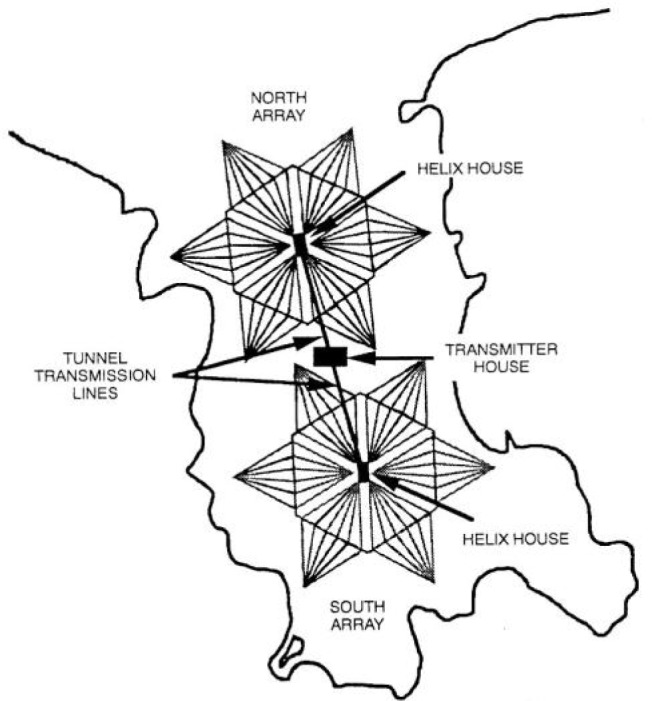
U.S. Navy radio station NAA, Cutler, Maine.

While Brown was working on the antenna system design, the U.S. Navy was also building a new radio station, in Cutler, Maine, along the Atlantic coast. This station, soon to become the most powerful in the world, had two huge antennas designed for operation in the VLF range. The antennas each consisted of six diamond-shaped arrays arranged in the shape of a radial star, delivering efficiencies previously unheard of in VLF stations and fed by a two-megawatt transmitter. A view of the Cutler station is shown in Fig. 8. The station commenced operations in 1961 with the call letters NAA. In both Brown’s earlier paper [35] and the NAA design [36, 37] the salient characteristic is the diamond shape of the individual antenna “panels”. This configuration would greatly influence Brown’s antenna design for the new NBS stations.

**Fig. 9:**
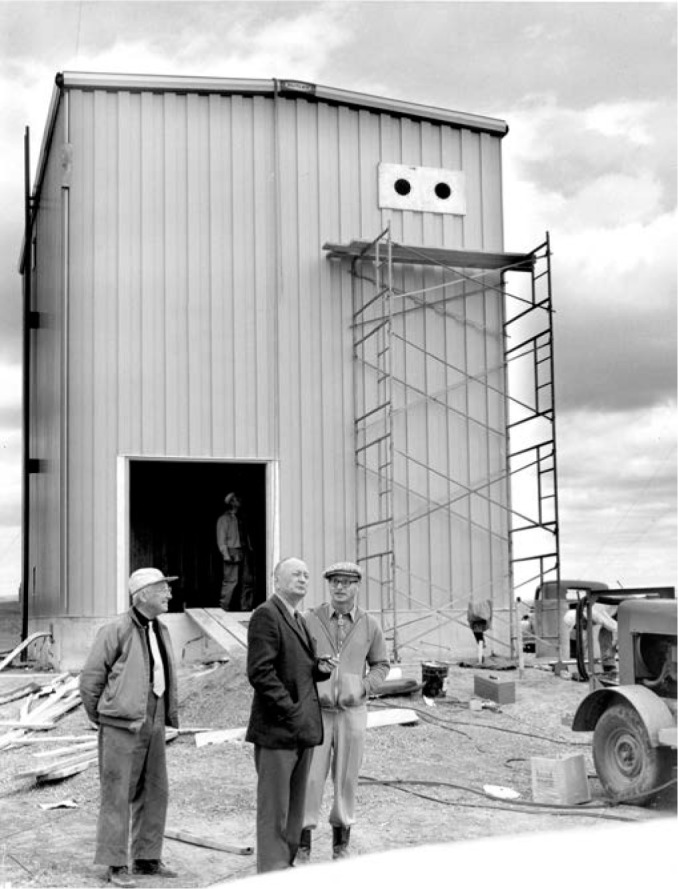
The construction phase of one of the helix houses at the Fort Collins site.

Brown’s plan called for two diamond shaped antennas ranging northwest to southeast across the property. The eight guyed steel towers, four for each array, would be 122 m tall. Each array would be 579 m long and 229 m wide. The panels, known in antenna parlance as capacitance hats or top hats, were made of eight aluminum cables running the length of the diamond. Across the center of the diamond from side to side ran a larger cable, spacing the longitudinal cables along its length. The panels were insulated from the grounded towers, and connected to concrete counterweights at the base of each tower. Each array had a vertical down lead that dropped from the center of the panel to a helix house, which contained antenna matching equipment and the connection to the transmitter. Figure 9 shows one of the helix houses during its construction, with Brown standing in the left foreground of the photograph.

**Fig. 10:**
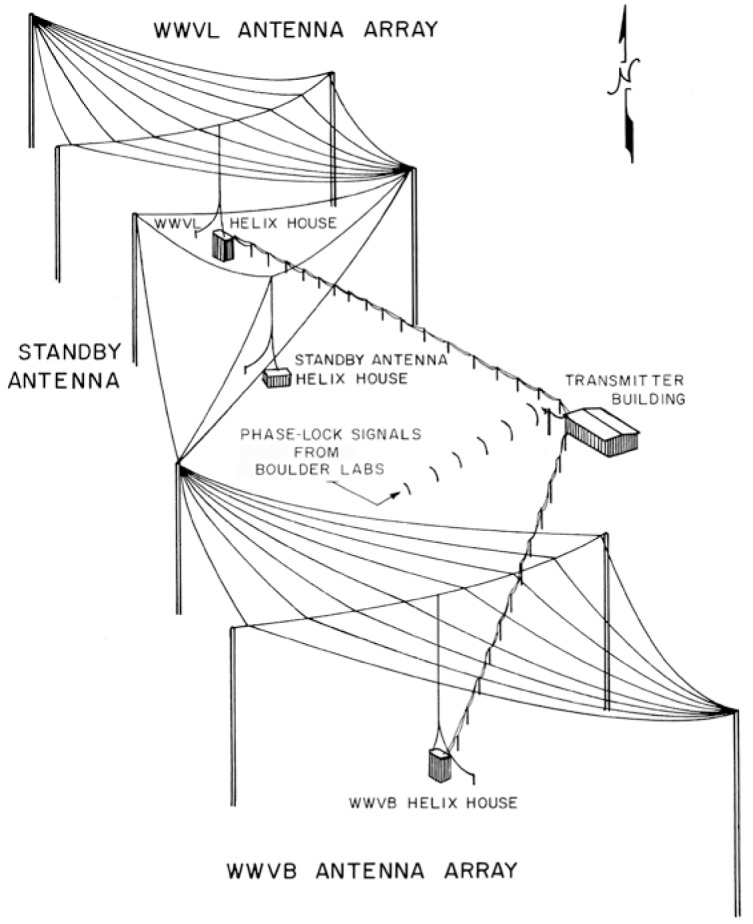
Drawing of WWVB and WWVL antenna arrays.

Buried underneath the arrays was a network of wires making up the grounded portion of the antenna, called the ground plane. The south antenna array would be used for the WWVB broadcast, and the north array for WWVL [7, 38]. The radiated efficiency of the antennas was estimated as 2.2 % at 20 kHz, and 24.6 % at 60 kHz [39]. An artist’s depiction of the two antennas made during the early days of their operation is shown in Fig. 10; they can also be seen on the left side of the model in Fig. 5, and marked as “Pilot Station” in Fig. 6. Note that Fig. 10 does not accurately show the orientation of the antennas with respect to each other (the south antenna appears offset to the west), but Fig. 6 does show the correct orientation.

**Fig. 11:**
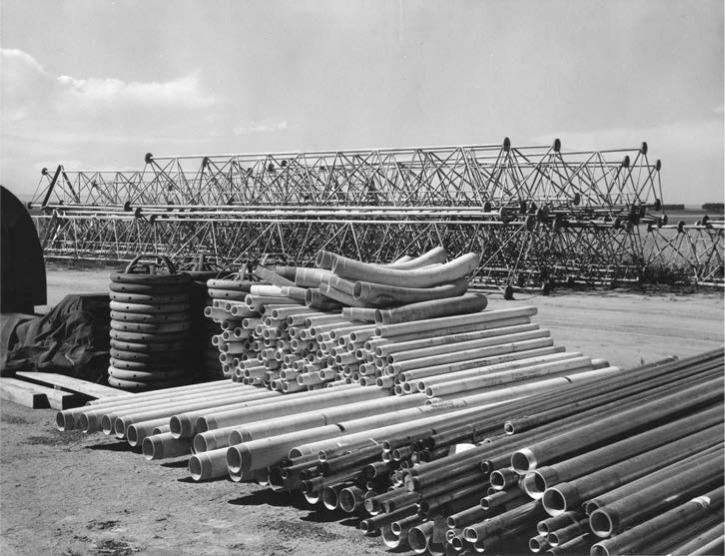
WWVB antenna parts on the ground prior to assembly, 1962.

**Fig. 12:**
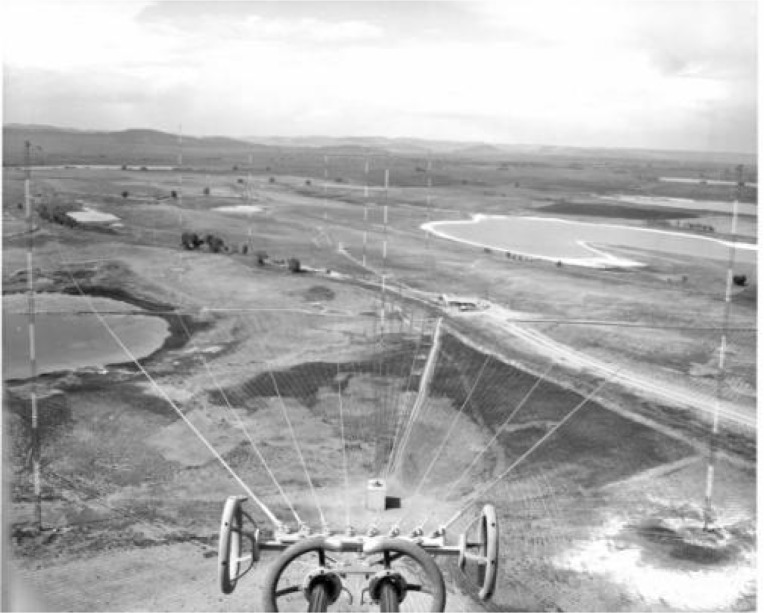
View of the antenna array from the top of Tower 1.

Construction began on the stations in July 1962 [33, 40], with placement of concrete anchors and foundations for the towers. The towers themselves were next, shipped in sections by rail to the nearby town of Wellington, Colorado and transported to the site by truck. The antenna cables and fittings arrived as well as the insulators and guy cables. Figure 11 shows various antennas and parts arranged on the ground prior to assembly. Figure 12 shows the view of both antenna arrays from the top of Tower 1. Careful inspection of the photograph will reveal one of the helix houses (center, near bottom) and the radial lines of the ground plane. An even closer inspection will reveal that there are nine towers in the photograph including the one in the foreground, even though only eight were included in the north and south arrays. The extra tower, known as Tower 9, was the standby antenna for both WWVB and WWVL and stood about 22 m taller than the other towers. Built in 1964, Tower 9 was disassembled and removed from the site in the early 1980s by a private contracting company. Sadly, a young workman was killed during the demolition process when a portion of the tower collapsed.

The original transmitters used at the new site were several refurbished and heavily modified ex-military transmitters, formerly known as model AN/FRT-6. Both WWVB and WWVL used versions of these transmitters.

**Fig. 13:**
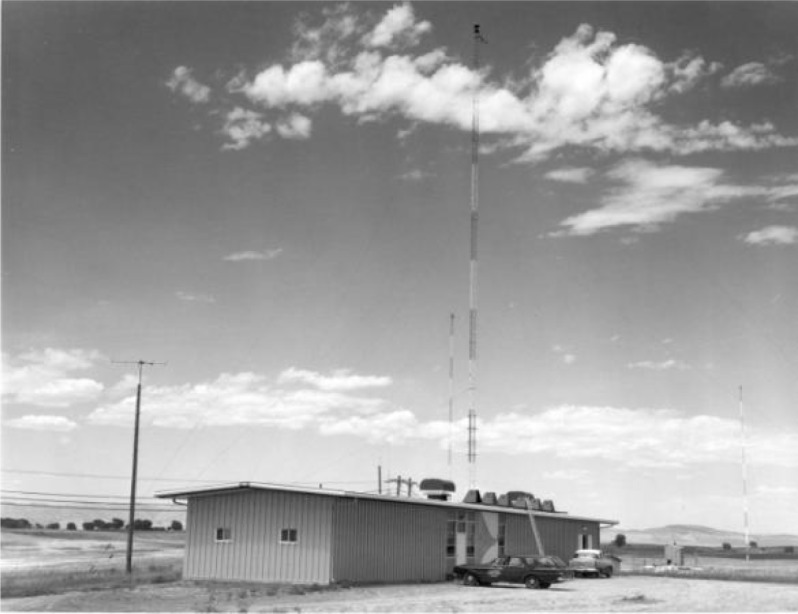
The original WWVB transmitter building in Fort Collins, completed in July 1963.

**Fig. 14:**
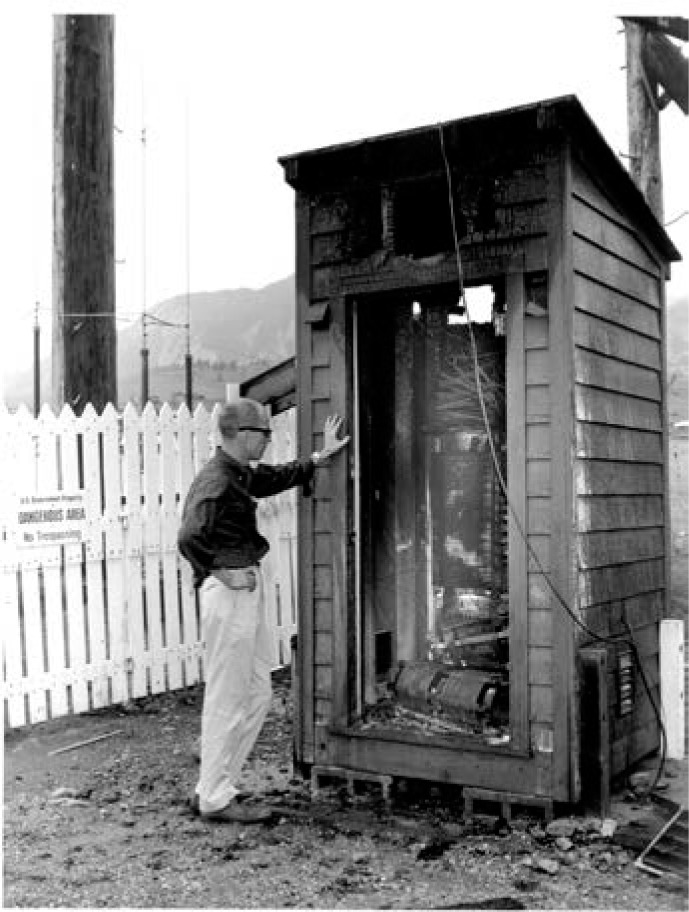
The experimental WWVB station in Boulder was destroyed by lightning shortly before the Fort Collins station went on the air.

The buildings and facilities in Fort Collins were completed in July 1963. The original building that housed the WWVB transmitters is shown in Fig. 13. Ironically, in mid-June, shortly before the completion of the new site, much of the equipment of the experimental WWVB station in Boulder was destroyed by a fire caused by a lightning strike on the antenna (Fig. 14). This event took the experimental 60 kHz station permanently off the air slightly earlier than anticipated [41].

**Fig. 15:**
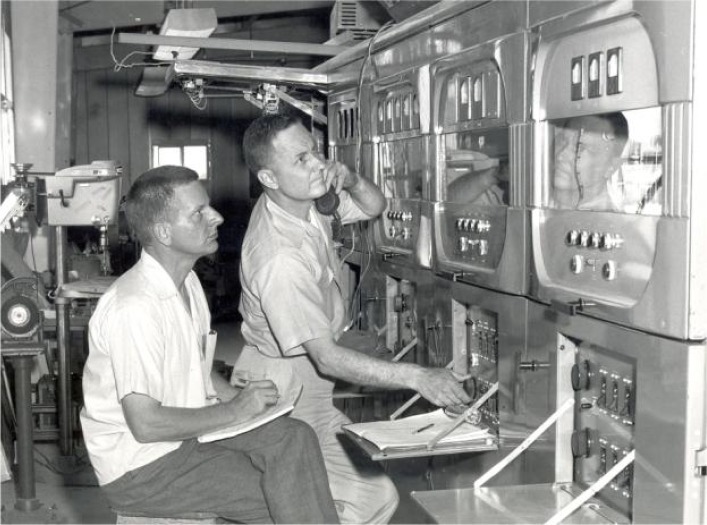
David Andrews (left) and Robert Oase (right) tuning the WWVB antenna.

Like Yankee Doodle Dandy, the new WWVB station was “born on the fourth of July”. The station began broadcasting from its new facilities at 5:00 PM local time on July 4, 1963 (0000 hours, July 5, Coordinated Universal Time) [27, 41]. The original radiated power was 4 kW. WWVL began transmissions in August 1963 (the exact date appears to be unpublished but may have been August 6^th^), radiating 500 W [42]. Figure 15 is a photograph of David Andrews and Robert Oase of NBS tuning the WWVB antenna to resonance about two weeks after the station went on the air. Equipment was later installed that tuned the antenna automatically.

Special dedication ceremonies for the new radio stations were held on August 13, 1963 at the NBS Boulder Laboratories, during the Symposium on Ionospheric Propagation of Low Frequency Waves. J. M. Richardson, the chief of the NBS Radio Standards Laboratory, chaired the dedication session, and NBS director A. V. Astin spoke. The dedication was followed by a series of technical talks about the new stations, after which buses took the symposium attendees to Fort Collins for a tour of the new site [32].

**Fig. 16:**
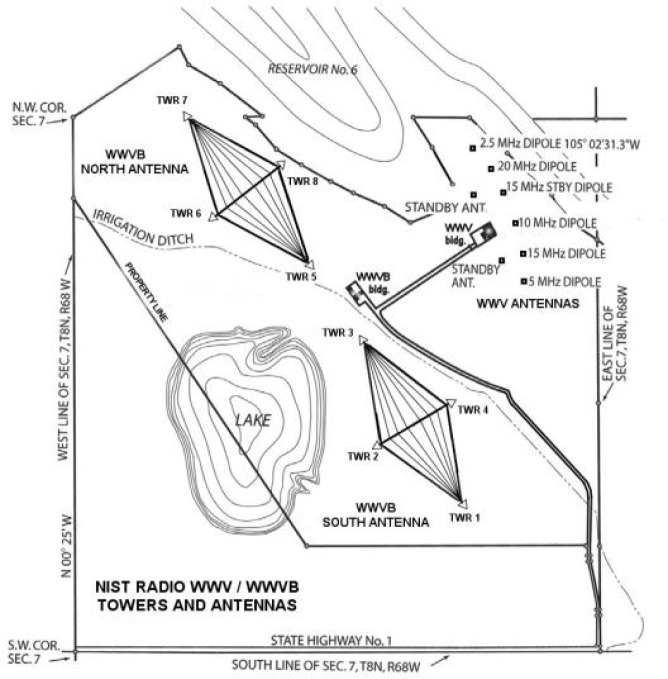
Map of the WWVB and WWV site, showing locations of antennas and transmitter buildings.

In July 1964, it was announced that WWV would be moved from Maryland to Fort Collins to share the WWVB/WWVL site, and Congress appropriated some $970,000 to provide for the move. WWV went on the air from Fort Collins on December 1, 1966 [17]. Figure 16 shows a map of the station site as it remains today, showing the eight WWVB towers in the north and south antenna arrays, the locations of the separate transmitter buildings for WWVB and WWV, and the locations of the WWV antennas.

## 10. WWVB Adds a Time Code

For approximately two years after going on the air in Fort Collins, WWVB’s only purpose was to serve as a standard frequency and time interval station. No time-of-day information was broadcast. The station broadcasted a 60 kHz sine wave that was largely unmodulated, with the exception of five cycles (83.3 µs duration) of 1000 Hz double-sideband amplitude modulation that were used to mark seconds [43]. In spite of the second markers the primary application for the station was, as it would remain until the 1990s, to serve as a frequency reference.

A digital time code that would allow receivers to recover the time-of-day was added to the WWVB broadcasts on July 1, 1965. The time code, designed by NBS engineer David Andrews [44], initially had very few users, although it would eventually make low-cost radio controlled clocks commonplace (Sec. 16). Although shortwave station WWV had added a time code in 1961 [45], WWVB was the first LF station to add a digital time code. The German station DCF77, which went on the air in 1959, did not add its widely-used time code until 1973 [46].

The time code transmitted information in a format known as binary coded decimal (BCD) where four binary digits (bits) were required to transmit one decimal number. Bits were modulated by simply lowering and raising the power of the 60 kHz carrier. The carrier power was dropped at the start of each second to send an on-time marker (OTM) for clock synchronization. Originally, the carrier power was dropped by 10 dB [47]. It remained this way until January 1, 2006, when the depth of the carrier phase modulation was increased to 17 dB [48] based on a suggestion from Casio, a manufacturer of radio controlled clocks. The increase in the power drop made the OTM easier to detect.

**Fig. 17:**
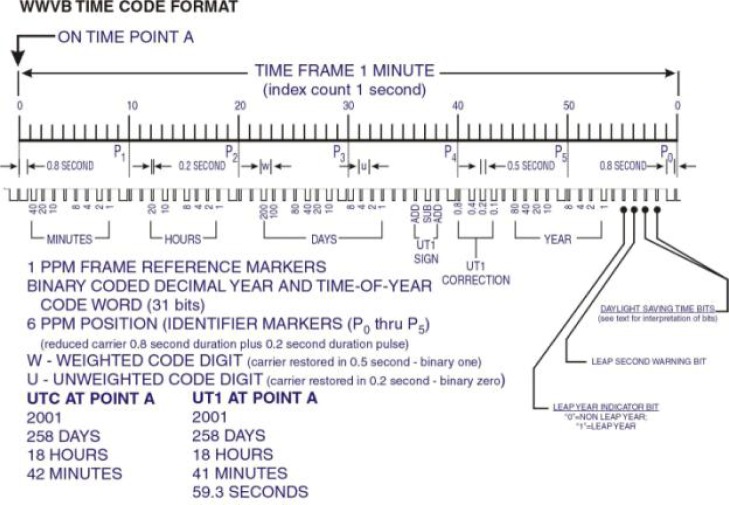
The WWVB “legacy” time code.

After the OTM is sent, the power is held low for 0.2 s to send a binary zero or for 0.5 s to send a binary one. This form of modulation is sometimes called amplitude shift keying, but is more properly referred to as pulse width modulation by reduced carrier transmission. This is because the information contained in the signal is demodulated by looking at the pulse widths (duration) rather than by looking at the amplitude. The bits are sent at the glacial rate of one bit per second, and a full minute is required to send a complete time code. The time code format (Fig. 17, [49]) has changed only slightly throughout the years, and is still utilized by nearly all WWVB products to recover time-of-day. However, an enhanced phase modulated time code was added to the broadcast in 2012 without disturbing the “legacy” time code, as is discussed later in Sec. 17.

WWVB has seldom been used for precise time synchronization applications (at a level better than 100 µs, or six 60-kHz cycles), largely because the exact OTM is not easy for receivers to identify [7]. However, in 1968 Andrews published a study where the received WWVB OTM was measured in Boulder. The uncertainty limitation for time transfer was identified as ±40 μs, or about ±2.5 cycles [50].

## 11. WWVL Goes Off the Air

After the plans to develop a worldwide frequency station at 20 kHz were scrapped (Sec. 8), the interest in WWVL waned. To be sure, the plans did not go away immediately – they were discussed well into the 1960s. However, as engineer John Milton noted in an NBS report of WWVB and WWVL field strength measurements – “In 1968, it became apparent that the financial resources of NBS would not support such a venture. Also work in the timing area by other government agencies in the fields of LF and VLF broadcasting, satellites and portable clocks made any NBS effort toward a higher power station scientifically questionable. It was then decided that NBS would officially withdraw ….” [51].

Milton was undoubtedly referring to the LF and VLF radionavigation systems like LORAN-C and Omega that were by this time widely used as frequency and time references and which each had a large network of stations that gave them an advantage over the single Fort Collins site. Satellites were an even larger factor in the decision to stop pursuing a worldwide VLF station. Satellite signals could cover large areas of the Earth with very little power, and the signal paths were exceptionally stable. NBS had already begun time synchronization experiments via satellite at the time of Milton’s report [52].

The radiated power of WWVL, which originated at 500 W at the Fort Collins site, was increased to 2 kW in 1966 [47] but remained there for the lifetime of the station. Within a few years, NBS began referring to the station as experimental, rather than as a continuous service. The station was used to study VLF time synchronization capabilities by utilizing a time-shared multi-frequency concept to conserve bandwidth. On November 4, 1969 the station began alternating between frequencies of 20.0 kHz, 19.9 kHz, and 20.9 kHz every 10 seconds [53]. However, users were informed that the format and frequencies were subject to change to meet the needs of the particular experiment being conducted. Users were encouraged to “explore alternative solutions to their needs” and the station operation became intermittent on January 1, 1972 [54]. WWVL was permanently turned off on July 1, 1972, less than nine years after going on the air in Fort Collins.

The north antenna array used for WWVL was left standing. It would remain essentially unused until the 1990s, when it was refurbished and became part of the WWVB transmission system (Sec. 15).

## 12. Controlling WWVB Frequency and Time

The purpose of WWVB has always been to deliver, by radio, the U. S. national standards of frequency and time to the general public. As previously noted, the actual national standard is located in Boulder, some 80 km away from the Fort Collins site. At this point in our narrative, it is worthwhile to describe the various methods used since 1963 to keep the station clock in Fort Collins in close agreement with the reference clock in Boulder.

The original method of controlling the WWVB frequency in Fort Collins was to use a phase-locked servo system where a control signal was broadcast from Boulder and received at the station site. This method was devised by Chesley Looney of NASA [55] and was first implemented by NBS to control the frequency of WWVL while it was located in Sunset, Colorado. The 20 kHz signal from WWVL was received in Boulder, where it was compared to the national standard. Then, a 50 MHz frequency modulated signal was sent from a Boulder transmitter back to the station site. The signal from Boulder contained the information necessary to correct the phase. When received at the station, it was used to drive a mechanical, two-phase servo motor that “turned the gears” and adjusted the station’s oscillator, keeping it within one or two parts in 10^11^ of the national standard [56].

**Fig. 18:**
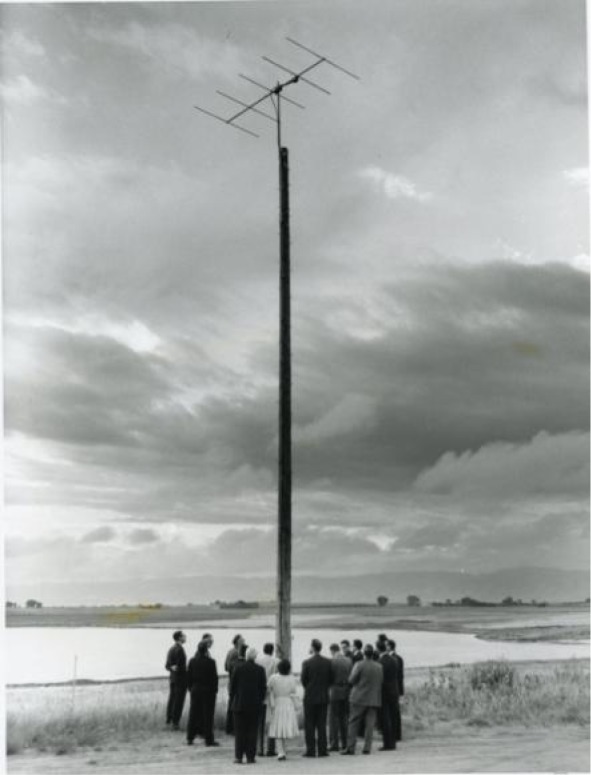
Antenna used to receive phase corrections from Boulder. Photograph taken at WWVB / WWVL station dedication, August 13, 1963.

**Fig. 19:**
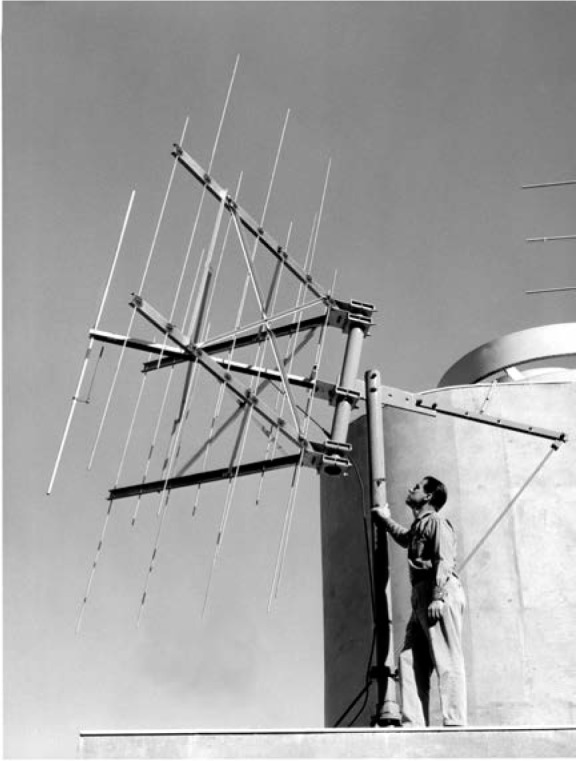
A 50 MHz transmitting antenna on the roof of the NBS laboratories in Boulder. This antenna was used to send phase corrections to the radio stations in Fort Collins.

When the system was implemented in Fort Collins, the signals of both WWVL and WWVB were received and measured in Boulder. When the 50 MHz signal transmitted from Boulder was received in Fort Collins it was split into three components – the reference phase information, the 20 kHz phase error information, and the 60 kHz phase error information [42]. Figure 18 shows a photograph taken at the dedication ceremony of the 40 ft antenna mast, topped by a Yagi antenna, that was used to receive the 50 MHz signal. The group of scientists at the bottom of the mast included NBS director A. V. Astin. Figure 19 shows the 50 MHz transmitting antenna on the roof of the NBS laboratories in Boulder. It is interesting to note that during this era the LF and VLF signals from Fort Collins, phased locked to Boulder, were received in Hawaii and Maryland (then the home of WWV) and used to control the frequency of the shortwave stations WWVH and WWV [27].

The continuous phase locked loop between Boulder and Fort Collins worked fairly well most of the time, but its performance was limited by short term phase errors due to sky wave interference. Thus, a replacement method was sought [57]. A new method, implemented in May 1968, involved common-view observation of television signals, a method first described by Tolman in Czechoslovakia [58]. The NBS implementation of this method utilized the reception of television signals broadcast from Lookout Mountain, which overlooks the city of Golden, Colorado south of Boulder. These signals provided television coverage to the entire Denver, Colorado metropolitan area. Television sets at Boulder laboratories and the radio station site were tuned to the same television program being broadcast from Lookout Mountain. Time interval counters in both Boulder and Fort Collins were started with pulses from the local clock, and stopped with pulses extracted from the television broadcast. Then, the difference between the Boulder reading and Fort Collins were compared to each other. If the Boulder clock agreed with the Fort Collins clock, the difference between the two readings would equal the difference between the two path delays, (*TV delay to Boulder*) – (*TV delay to Fort Collins*), which was about 63.5 µs. If the Fort Collins clock was ahead of or behind the Boulder clock, it was manually adjusted until the desired delay value was reached. This method allowed the Fort Collins clock to be continuously held to within about ±0.5 µs (500 ns) of the national time standard [57, 59, 60].

Common-view television measurements were eventually replaced by higher accuracy common-view measurements via Global Positioning System (GPS) satellites, a technique that NBS helped pioneer shortly after the launch of the first GPS satellite in 1978 [61, 62]. The Boulder clock and the Fort Collins clock were each compared to the same GPS satellite at the same time. The Boulder and Fort Collins measurements were exchanged by computers connected to telephone modems, and in later years, by computers connected to the Internet. The difference between the two measurements was the difference between the Boulder and Fort Collins clocks, as the clock onboard the GPS would fall out of the equation. This method made it routine to keep the cesium clock at the station within ±0.1 µs (100 ns) of the national time standard in Boulder, and to agree to within 1 × 10^−13^ in frequency.

Common-view GPS measurements are still used to compare Fort Collins time to Boulder, but an ensemble time scale that outputs the weighted average of four cesium clocks was installed in Fort Collins and became the reference for WWVB in February 2005 [63]. The stability of the time scale now allows the station time to be typically kept within ±0.02 µs (20 ns) of the national time standard in Boulder, and to agree to within 1 × 10^−14^ in frequency.

## 13. Scientific Applications of WWVB

In the first few years after WWVB and WWVL moved to Fort Collins, the new stations were routinely utilized by NBS personnel for scientific and research purposes. Attempts were made to utilize the stations, in particular WWVL, for international comparisons of atomic clocks [64]; although LORAN-C (and eventually GPS) would prove to be much better suited for that role. Propagation studies of WWVL were conducted to look at the magnitude of the diurnal phase fluctuations at various distances [65], and an extensive field strength study was conducted for both stations [51].

Published research conducted outside of NBS indicates that WWVB was utilized during its early years for a variety of scientific applications, including the correlation of seismic events [66, 67], astronomical observations; including the measurement of radio emissions from Jupiter [68], studies of underwater sound propagation [69], and studies of the attenuation of VLF and LF radio signals as they pass through the Earth’s surface [70].

Within a few years after WWVB went on the air in Fort Collins, two watershed events changed the direction of the station’s future, hastening its transition from a combination of radio research facility and metrology service, to a full-time metrology service. The first event occurred in October 1965, when after some 20 years of being an integral part of NBS, the Central Radio Propagation Laboratory (CRPL) was transferred to a new scientific agency called the Environmental Science Services Administration (ESSA). Within ESSA, the name of CRPL was changed to the Institute for Telecommunication Sciences and Aeronomy (ITSA). As a result of the transfer, NBS lost a number of radio engineers and some of its radio research facilities to ITSA, which stayed at the same location in Boulder, Colorado. This made NBS less likely to engage in radio research and propagation studies and more likely to focus on using its radio facilities to support measurement and primary standards work [1]. Of course, WWVB, as well as its sister stations WWV, WWVH, and WWVL, were not transferred to ITSA. They stayed with NBS and remained a key part of its basic standards work.

The second event occurred in 1967 when the Time and Frequency division of NBS was formed at the Boulder laboratories. It was a logical year to form a new division, because 1967 was when the second was internationally agreed upon to be based on an atomic transition (specifically as 9,192,631,770 energy transitions of the cesium atom). The redefined second officially began the era of atomic timekeeping. NBS had engaged in many different time and frequency activities for decades, including the development of atomic clocks as well as the operation of the radio stations, but these activities had previously been spread across several groups. Everything was now consolidated into one division [1]. Within the new division, the primary role of WWVB, as it remains to this day, was to serve as a time and frequency service – a freely available signal source that delivered standard frequency and time signals, referenced to the national standard, to the American public. Accuracy, as always, remained important, but reliability became almost equally important. During the years that followed, many commercial products, including disciplined oscillators and radio controlled clocks, were manufactured to receive WWVB and take advantage of this free U. S. government service, as described in the following sections.

## 14. WWVB Disciplined Oscillators

Starting in the 1960s, WWVB disciplined oscillator products were available commercially. These products consisted of a WWVB receiver and antenna, a phase detector/comparator, and a local quartz oscillator. The phase comparator was used to measure the difference between the local oscillator and the incoming WWVB signal. A correction was then issued to the local oscillator to keep it phase locked to WWVB. Some devices allowed users to connect another oscillator, which could then be calibrated by comparing it to the oscillator that was phase locked to WWVB [71]. The uncertainty of a WWVB disciplined oscillator was related to the WWVB signal quality, which meant that it was often a function of the receiver’s distance from the transmitter. However, a frequency uncertainty of less than 1 × 10^−11^ could often be obtained with one day of averaging in most parts of the United States.

**Fig. 20:**
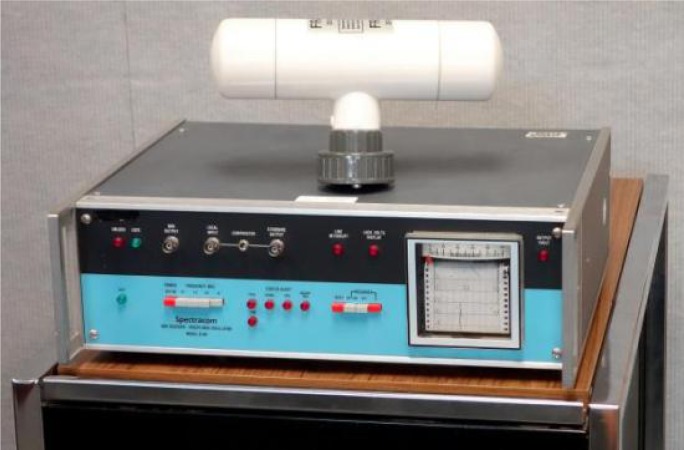
Spectracom 8164 WWVB Disciplined Oscillator (A. Novick, NIST).

The manufacturers who once sold WWVB disciplined oscillator products include Fluke, Hewlett-Packard, Spectracom, Tracor, and True Time/Kinemetrics. Spectracom, located in East Rochester, New York, probably sold the most units. The Spectracom 8160 series of products were once commonly found in standards and calibration laboratories, with Spectracom receiving a patent for their disciplined oscillator system in 1985 [72]. Figure 20 is a photograph of the Spectracom 8164.

Most WWVB disciplined oscillator products had disappeared from the marketplace by the year 2000, replaced by GPS disciplined oscillators, which typically had uncertainties that were one or two orders of magnitude smaller than the WWVB products, and that could work reliably anywhere on Earth. The small number of WWVB disciplined oscillator products that still remained in operation became obsolete in October 2012, when a phase modulated time code was added to the WWVB carrier (Sec. 17).

## 15. WWVB Power Upgrade

As noted earlier, WWVB began operations at the Fort Collins site with a radiated output power of 4 kW [42]. This was increased to 7 kW shortly after the station went on the air [43]. NBS regularly published guides for users of its broadcast services, and the radiated output power was listed as 10 kW in the 1966 guide [47], increasing to 12 kW by 1967 [73], to 16 kW in 1969 [74], and finally settling at 13 kW in 1972 [54], where it remained for many years. The 13 kW power level was usually sufficient for high quality receivers with antennas mounted outdoors. Even then, however, reception was not always reliable, especially in regions such as the northeastern states or southern Florida.

WWVB continued broadcasting throughout the 1970s, 1980s, and early 1990s with only minor modifications made to its format or equipment. The number of users of the signal was relatively small; mostly calibration laboratories who operated WWVB disciplined oscillators, such as the devices described in Sec. 14. The limitations of the aging transmitting equipment at WWVB became increasingly apparent as the years passed. The situation came to a head on February 7, 1994 when a heavy mist froze to the antenna, and the antenna tuning system could not compensate, shutting down the WWVB broadcasts for about 30 hours [75]. After reviewing the available options, it was decided that a redesign of the entire WWVB transmitting system was necessary [7].

During the discussions about redesigning WWVB, the decision was made to make WWVB more “relevant” by substantially raising its power level. It was obvious that WWVB could play a larger role in the time and frequency community if its signal was easy to receive with low cost equipment. In Europe, low cost radio controlled clocks were beginning to appear, designed to synchronize to stations such as MSF in the United Kingdom and DCF77 in Germany [46]. These stations were very similar to WWVB, but had a much smaller coverage area to service. As a result, European customers were able to purchase radio controlled alarm clocks, wall clocks, and wristwatches that worked indoors with small antennas hidden inside their case. These clocks had an advantage over satellite controlled clocks, which could not reliably work indoors without a view of the sky. Don Sullivan, then the chief of the NIST Time and Frequency division, believed that these same products being sold in Europe could be offered in the United States if the WWVB signals were made strong enough to blanket the country. His reasoning proved to be correct, as we shall see in the next section.

Beginning in October 1994, consultants and engineers from the U.S. Navy’s LF/VLF support group were hired by NIST (the agency’s name changed from NBS to NIST in 1988) to evaluate the existing WWVB system and to propose changes. Their reports [31, 38] suggested that although the antennas themselves were in reasonably good shape, the transmitters and matching equipment should be completely redesigned and new or upgraded equipment installed. The project progressed in phases over the next several years, resulting in the transfer of modern LF transmitters (AN/FRT-72) and other equipment from recently decommissioned Navy facilities to NIST. New station staff members were hired who had previous experience with the acquired systems and equipment. Contractors normally employed by the Navy for LF work were hired to design a new broadcast control system that fully utilized the assets of the existing station [7].

A formal announcement that the WWVB power was to be increased was made during 1996. The transmitters were received and installed that same year. The station modernization took place throughout 1997, and by December 1997, an interim stage of the upgrade was completed and the radiated output power was increased to about 27 kW. By August 5, 1999, the upgrade was complete. The new WWVB configuration used two transmitters operating into two antennas that simultaneously broadcast the same 60 kHz signal. The second antenna was the north antenna, which had been essentially unused since WWVL had gone off the air in 1972, except for a few instances when it was used as an emergency backup to the south antenna [32]. When used individually, the north antenna was found to be about 56 % efficient and the south antenna had an efficiency of about 54 %. When the two antennas were combined into one array, the efficiency increased to almost 69 %, allowing 50 kW to be radiated with only about 37 kW input to both antennas [7]. This represented about four times more power (6 dB) than the pre-upgrade configuration.

**Fig. 21:**
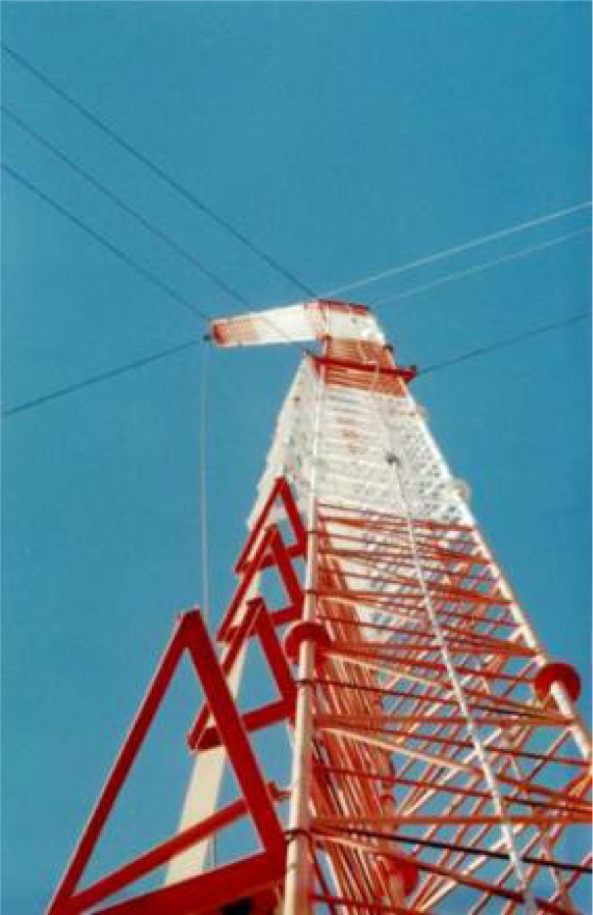
A view from the ground of Tower 7 after the 2001 insulator pin failure.

The redesigned station suffered a temporary setback on February 27, 2001, when the uppermost 30 m of one of the 122 m high WWVB towers was severely bent due to the failure of an insulator pin (Fig. 21) [76]. The failure occurred with Tower 7, one of four towers in the north antenna. The required repairs were extensive, and for the next several months the station operated with only the south antenna, although increasing the input power to the south antenna allowed the station to radiate about 37 kW. Transmission from both antennas resumed on May 14, 2001, and the station returned to normal operation on June 14, 2001 when the repairs were completed. On September 29, 2003 a less extensive failure again occurred to Tower 7, but this time it was still possible to tune the north antenna and keep it operational most of the time. Even so, the station operated with only the south antenna for brief periods until August 2004.

The transmission system at WWVB has continuously undergone small improvements in recent years to optimize the output power. At this writing (2013) the input to each antenna is about 51 kW. The current efficiency of the combined antenna array is estimated at 68.8 %, resulting in an effective radiated output power of about 70 kW [77].

## 16. WWVB Radio Controlled Clocks

For more than three decades after going on the air in Fort Collins, WWVB had gradually been transitioning from being a reference source for frequency to being a reference source for time-of-day. This transition was greatly accelerated after the power upgrade was announced in 1996. As noted in Sec. 10, WWVB began broadcasting a time code on July 1, 1965. Even so, radio controlled clocks (RCCs) did not instantly appear, and the first application of the new time code was to add time markers to strip chart recordings, particularly for the timing of seismic events [66].

A few years after the time code was added, a small number of clocks appeared that could automatically decode and display WWVB time. The first commercially available RCC was probably the Develco 3391, which was providing accurate time for the electric power industry by 1969 [78]. Some hobbyists began building WWVB RCCs in the early 1970s based on a design published in *Radio Electronics* [79, 80], and a few commercial models became available throughout the 1970s and 1980s, built by manufacturers such as Spectracom and True Time/Kinemetrics. For the most part, however, these clocks were unknown to the general public, and their use was limited to scientific and industrial applications. They usually required an outdoor antenna. During the pre-microprocessor days they also required a substantial amount of electronic circuitry, which made them expensive to manufacture.

By the time of the power upgrade announcement in 1996, it was much more feasible for low-cost RCCs to be developed. Low cost microprocessor units made it easy to decode the signal under less than optimal conditions. The circuits used very little power and could be battery operated, often requiring only a single AA cell. Small ferrite bar antennas were available that could be embedded inside the plastic case of a RCC. As a result, WWVB RCCs began entering homes and offices shortly after the 1996 announcement of the station power upgrade, with some models appearing even before the station began broadcasting higher power levels.

**Fig. 22:**
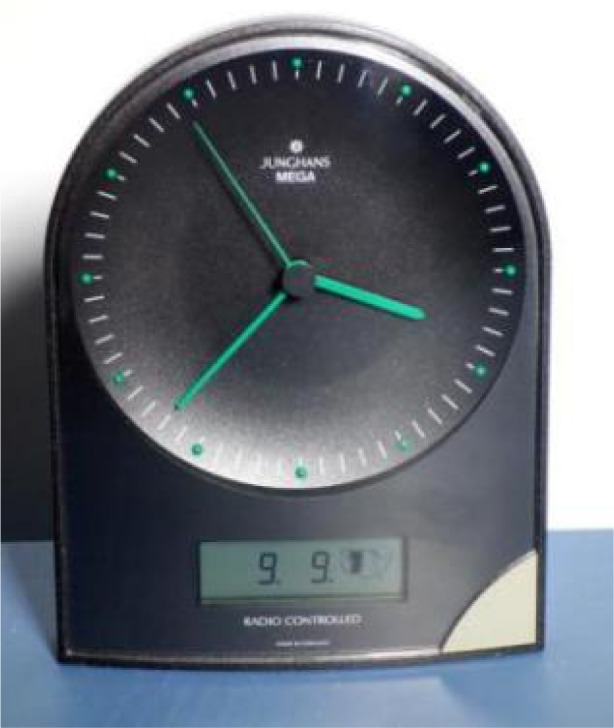
The Junghans MEGA, introduced in the United States around 1996, was probably the first low cost WWVB radio controlled clock (A. Novick, NIST).

The first low-cost WWVB RCC intended for home use was probably manufactured by the German company Junghans, which began selling a desktop WWVB clock (Fig. 22) in the United States in 1996. Earlier versions of this clock were sold in Germany for the reception of DCF77. Semiconductor manufacturers such as Telefunken in Germany and Atmel in the United States soon released single-chip WWVB receivers. A steady stream of WWVB wall clocks, desk clocks, and wristwatches [81] were introduced, and new products continue to be released. WWVB RCCs are now commonly found in U. S. department and discount stores, with prices sometimes dropping below $10 USD, making NIST time available to nearly all of the U. S. population. Manufacturers and resellers of WWVB RCCs have included Acurite, Arcron-Zeit, Casio, Emerson, Howard Miller, Lacrosse Technology, Oregon Scientific, Primex, Radio Shack, Sangean, Seiko, Sharp, Sky Scan, Timex, and many others.

**Fig. 23:**
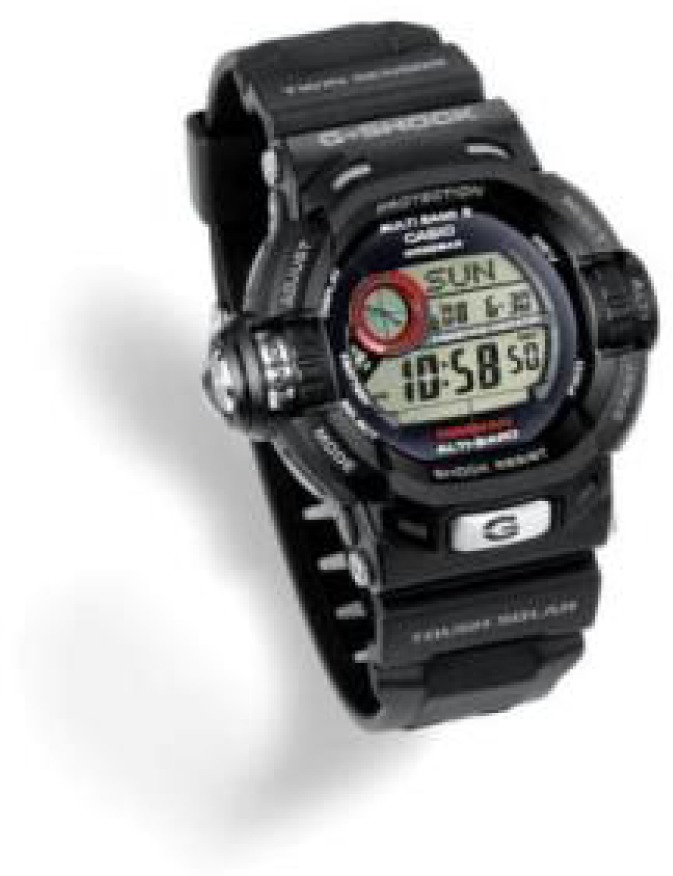
A Casio “Multi Band 6” wristwatch. Introduced in 2008, these devices can synchronize to LF time signals from China (68.5 kHz), Germany (77.5 kHz), Japan (40 and 60 kHz), and the United Kingdom (60 kHz), in addition to the 60 kHz signal from WWVB (courtesy of Casio).

The time code format utilized by WWVB is not identical to the time codes of other LF time signal stations. However, it is similar, as are the transmission frequencies, which all fall in the range from 40 kHz to 80 kHz. This has led to “multiband” technology which allows some RCCs to synchronize throughout much of the United States, Asia, and Europe. Figure 23 shows a wristwatch that can synchronize to six different LF time signal stations, provided that it is within receiving range of the transmitter.

As expected, the many millions of WWVB RCCs in operation resulted in a large number of technical support questions being directed to the Time and Frequency Services group of NIST. To assist both manufacturers and consumers of RCC products, NIST published *Special Publication 960–14* in 2005 (revised in 2009) [82]. The guide is believed to have helped RCC manufacturers improve the quality and reliability of their products, and to help consumers better understand how the clocks work.

Consumers commonly ask about the accuracy of WWVB RCCs. Most RCCs synchronize to the WWVB time code once every 24 hours and are usually accurate to within ~30 ms at the time of synchronization, even if the clock is located at the far reaches of the station’s coverage area. However, in between synchronizations, an RCC is no more accurate than their internal quartz oscillator. If the frequency of the RCC’s quartz oscillator is accurate to within a few parts per million, ~3 × 10^−6^ (which is not difficult to achieve even with inexpensive quartz crystals), then the accumulated time error between synchronizations will not exceed about a quarter of a second. A time error that small often cannot be detected by a person viewing an analog or digital clock display. Thus, a WWVB RCC appears to always be exactly synchronized to NIST time if it can synchronize once per day [83].

## 17. Modulation Changes to Improve Radio Controlled Clock Reception

After the WWVB power upgrade was completed in 1999, RCCs generally worked well in the 48 states of the continental U. S., with intermittent reception even reported in Hawaii and Alaska during the nighttime hours. However, WWVB reception was often sporadic in the daytime hours and sometimes difficult even at night, particularly in the northeastern states and in southern Florida, which were both heavily populated areas. This was due to several factors; including the absorption of the sky wave signal by the ionosphere during the daytime hours, atmospheric noise from electrical storms and other sources, local and RF interference, multipath (ground wave/sky wave cancellation), and even interference from the British station MSF, which also transmits at 60 kHz across the Atlantic. This situation was improved somewhat by increasing the depth of the carrier amplitude modulation to 17 dB in 2006 [48], but it was believed that the only true solution was to build another LF time signal station in the eastern United States that operated on a different frequency, which was expected to be 40 kHz.

NIST investigated several site locations for a new station including Annapolis, Maryland, and Greenville, North Carolina, and pursued various funding opportunities. After several years of searching for resources, federal funding was appropriated through the 2009 American Recovery and Reinvestment Act (ARRA) for a new station at the Redstone Arsenal in Huntsville, Alabama. Redstone Arsenal is operated by the U.S. Army, and includes several research and testing activities from the Department of Defense, NASA (Marshall Space Flight Center), and other Federal agencies. Representatives of some activities at Redstone expressed concern about potential radio frequency interference from the proposed high power LF station, and after extensive discussions it was determined that the new LF station should not be built at Redstone. By law, the ARRA funds had to be spent within a limited time frame, which did not provide the opportunity to make plans for a different location. The ARRA funding was reprogrammed with agreement by Congress and the Administration to help fund the construction of a new advanced research and measurement laboratory on the NIST Boulder campus [84].

After the unsuccessful attempt to build a new station, the attention shifted to finding new ways to making the existing WWVB signal from Fort Collins easier to receive. It was decided to add a phase modulated (PM) time code to the 60 kHz carrier that would not affect the legacy pulse width modulated time code. This approach had formerly been implemented in 1988 by the LF time station DCF77 in Germany [85]. Clocks designed to receive the PM code would benefit from an increased signal-to-noise ratio, making the time code easier to decode, and thus making RCCs more reliable.

Through its Small Business Innovation Research (SBIR) program, NIST awarded a grant to Xtendwave, a company located in Dallas, Texas, in September 2010. The grant was for the design of time code generators and time code receivers for a PM time code that could be reliably received even during harsh signal conditions. In cooperation with NIST, Xtendwave designed the PM time code by utilizing a modulation scheme called binary phase shift keying (BPSK). This technique differentiates between a 0 and a 1 bit by shifting the phase of the 60 kHz carrier [86]. A 0 bit is transmitted when there is no phase shift, and a 1 bit is transmitted by inverting the phase of the carrier by 180º. The phase transition between each bit and the next one in the 1 bps (bit per second) PM frame occurs 100 ms after the carrier amplitude drop that indicates the start of a second.

The duration of the PM time frame is 60 s. It includes a synchronization word, a time word, and daylight saving time (DST) and leap second information. The time word, from which the year (excluding century), the date, and the hour and minutes may be extracted, is represented by a 26-bit minute counter that represents the number of minutes that have elapsed since this century began at 00:00 Coordinated Universal Time on January 1, 2000. The 26-bit time word is converted to a 31-bit Hamming code by the addition of five error correcting bits. This allows the time code receiver to correct one error and to detect up to two errors in the time word [87].

The PM time code system was designed to be scalable, and allows for receivers experiencing different reception conditions to use the received signal differently. In particular, it allows for the accumulation of received energy over multiple one-minute frames, to provide for a corresponding signal gain in the receiver (i.e., reception for a whole hour may provide a gain of 60, or 18 dB, with respect to reception for a single minute) [86]. This can greatly increase the robustness and reliability of RCCs designed to utilize the PM time code, making them, for example, much more likely to be able to synchronize in the daylight hours. This is expected to lead to WWVB receivers being embedded in many types of products where they are currently not found – including industrial control systems, automobiles, kitchen appliances, and sprinkler systems. Xtendwave has designed [88, 89] and is manufacturing WWVB receiver integrated circuits, and other manufacturers are expected to eventually follow suit.

The format became permanent on October 29, 2012 after about one year of testing. The tests demonstrated that the new PM time code did not affect the many millions of low-cost RCCs already in operation that received the legacy time code. However, it did render the old WWVB disciplined oscillator products described in Sec. 14 obsolete. As a service to the few remaining users of these products, the PM time code was initially omitted from the broadcast twice daily for 30 minutes, beginning at noon and midnight Mountain time. This practice was discontinued on March 21, 2013 when the broadcasting of the PM time code became continuous.

At this writing (September 2013) the station is also experimenting with a fast PM time code that, if implemented, will allow clocks to synchronize every 10 s, as an alternative to the standard 60 s time code frame. The fast time code will not interfere with either the legacy pulse width modulated code or the standard PM time code. This will allow “intelligent” RCCs to be designed that select the time code best suited for the current receiving conditions.

## 18. Summary

The year 2013 marks the 50th anniversary of the NIST radio station WWVB broadcasts from Fort Collins, Colorado. As we have seen in this narrative, however, the actual story of WWVB began much earlier, when NIST’s predecessor, the National Bureau of Standards, began conducting early radio experiments. Throughout its long and colorful history, WWVB has demonstrated an almost uncanny ability to evolve and “reinvent” itself, by adapting old technology to meet the needs of new applications. It has now fully transitioned from a station whose primary function was to serve as a frequency reference, to one that provides daily time-of-day synchronizations for many millions of radio controlled clocks found in U. S. homes and businesses. One of the most popular and most relevant metrology services provided by NIST, WWVB should continue to serve the timekeeping needs of the American public for decades to come.

## Footnotes

1 Certain commercial equipment, instruments, or materials are identified in this paper to foster understanding. Such identification does not imply recommendation or endorsement by the National Institute of Standards and Technology, nor does it imply that the materials or equipment identified are necessarily the best available for the purpose.
